# Rare immune diseases paving the road for genome editing-based precision medicine

**DOI:** 10.3389/fgeed.2023.1114996

**Published:** 2023-02-08

**Authors:** Mara Pavel-Dinu, Simon Borna, Rosa Bacchetta

**Affiliations:** ^1^ Division of Hematology-Oncology-Stem Cell Transplantation and Regenerative Medicine, Department of Pediatrics, Stanford Medical School, Palo Alto, CA, United States; ^2^ Center for Definitive and Curative Medicine, Stanford University School of Medicine, Palo Alto, CA, United States

**Keywords:** CRISPR/Cas9, adeno-associated virus 6, base editing, prime and twin prime editing, hematopoietic stem and progenitor cells, RAG2 deficiency, *FOXP3*, IPEX syndrome

## Abstract

Clustered regularly interspaced short palindromic repeats (CRISPR) genome editing platform heralds a new era of gene therapy. Innovative treatments for life-threatening monogenic diseases of the blood and immune system are transitioning from semi-random gene addition to precise modification of defective genes. As these therapies enter first-in-human clinical trials, their long-term safety and efficacy will inform the future generation of genome editing-based medicine. Here we discuss the significance of Inborn Errors of Immunity as disease prototypes for establishing and advancing precision medicine. We will review the feasibility of clustered regularly interspaced short palindromic repeats-based genome editing platforms to modify the DNA sequence of primary cells and describe two emerging genome editing approaches to treat *RAG2* deficiency, a primary immunodeficiency, and *FOXP3* deficiency, a primary immune regulatory disorder.

## Introduction

Progress in developing new and curative therapies for rare monogenic diseases has dramatically improved in recent years. Specifically, in the past two decades, gene therapy-based precision medicine (Naldini, 2011; High and Roncarolo, 2019; Porteus, 2019) has shown remarkable promise and success in treating monogenic diseases in cases where the molecular aberrations leading to the disease pathology are defined. Among the ∼10,000 known rare monogenic diseases (Haendel et al., 2020), inborn errors of immunity (IEI), including Primary immunodeficiencies (PIDs) and Primary Immune Regulatory Disorders (PIRDs), are a growing class of rare diseases (Demirdag et al., 2021). PIDs and PIRDs affect 1:10,000 to 1,000,000 live births worldwide (Bousfiha et al., 2013; Tangye et al., 2020), with incidents as high as 1:500 in regions with consanguineous inheritance (Meshaal et al., 2019; Greenberg-Kushnir et al., 2020). The genetic cause of many PIDs (Notarangelo, 2010) and PIRDs is known, with testing and diagnosis being made available at birth. Early diagnosis is vital to enable management strategies, guide prognosis, and initiate life-saving treatments. As of 2022, there are 485 IEI (Tangye et al., 2022), each represented by unique gene mutations that partially or completely disrupt the normal development and function of the adaptive and/or innate immune system. Deep knowledge of IEI pathophysiology is necessary to establish safe and curative treatment approaches.

The decades-long effort to identify and modify pathogenic nucleotide variants in genomic DNA (Smithies et al., 1985; Rouet et al., 1994; Choulika et al., 1995; Jasin, 1996; Bibikova et al., 2003; Porteus and Baltimore, 2003; Porteus et al., 2003; Porteus and Carroll, 2005; Urnov et al., 2005; Grizot et al., 2009; Miller et al., 2011; Voit et al., 2014) has yielded a powerful technology—genome editing - based on inducing DNA double-stranded breaks (DSBs) or nicks to achieve defined genetic changes and restore normal gene function. The safety and efficacy of genome editing in human hematopoietic stem and progenitor cells (HSPCs) and T cells have been tested preclinically (Dever et al., 2016; Hubbard et al., 2016; Liang et al., 2017; Pavel-Dinu et al., 2019; Goodwin et al., 2020; Cromer et al., 2021; Gray et al., 2021; Sweeney et al., 2021; Vavassori et al., 2021; Wilkinson et al., 2021; Iancu et al., 2023) with some approved by Food and Drug Administration (FDA) for Phase 1 clinical trials in indications such as hemoglobinopathies (Porteus et al., 2022), congenital blindness (Maeder et al., 2019), and hearing loss (Botto et al., 2021). The therapeutic application of genome editing technology to correct gene dysfunction in IEIs has gained momentum, mainly due to the ability to preserve critical endogenous expression and regulatory features, which are indispensable for proper immune development and function.

This review focuses on the Clustered Regulatory Interspaced Short Palindromic Repeats (CRISPR) Cas9 nuclease-based genome editing toolbox and describes their applications in correcting genes regulating the immune system. We discuss the pre-clinical development of CRISPR/Cas9-based therapies for *RAG2*-SCID, a PID, and *IPEX,* a PIRD, which could benefit from genome editing to restore optimal immune function.

## Genome editing technology emerges as a therapeutic tool

Innovations in DNA sequencing technology combined with an understanding of how to stimulate and modulate DNA repair to levels compatible with clinical applications (Lattanzi et al., 2021), set the foundation for genome editing. Genome editing is the process of engineering changes in the DNA sequence of a cell. The modification ranges from a single nucleotide to inserting a full-length complementary DNA (cDNA) (Porteus, 2019). Significant advancements in genome editing came from discovering CRISPR/Cas9, a bacterial nuclease used as a defense mechanism against invading parasites (Gasiunas et al., 2012; Jinek et al., 2012). By designing a chemically modified 20-nucleotide RNA guide (Hendel et al., 2015) (single guide RNA; sgRNA) complementary to a pre-selected DNA sequence in the human genome (Mali et al., 2013), CRISPR/Cas9-sgRNA can now be programmed to recognize and induce DSBs, at most genomic locations, an event that recruits DNA repair machinery and prompts DNA sequence modifications (Yeh et al., 2019). The versatility (Gaudelli et al., 2017; Kim et al., 2017; Komor et al., 2017; Bak et al., 2018; Koblan et al., 2018; Rees and Liu, 2018; Anzalone et al., 2019; Jeong et al., 2020; Sakata et al., 2020; Sato et al., 2020; Zeng et al., 2020; Anzalone et al., 2022), efficacy (Genovese et al., 2014; Hoban et al., 2015; Dever et al., 2016; Schiroli et al., 2017; Kuo et al., 2018; Pavel-Dinu et al., 2019; Roman-Rodriguez et al., 2019; Goodwin et al., 2020; Rai et al., 2020; Cromer et al., 2021; De Ravin et al., 2021; Sweeney et al., 2021; Iancu et al., 2023) and specificity (Vakulskas et al., 2018) of the CRISPR/Cas9-sgRNA-based genome editing platforms have revolutionized our ability to recognize and correct disease-causing variants directly in the genome of primary human cells.

### Genome editing with a double strand break

**FIGURE 1:**
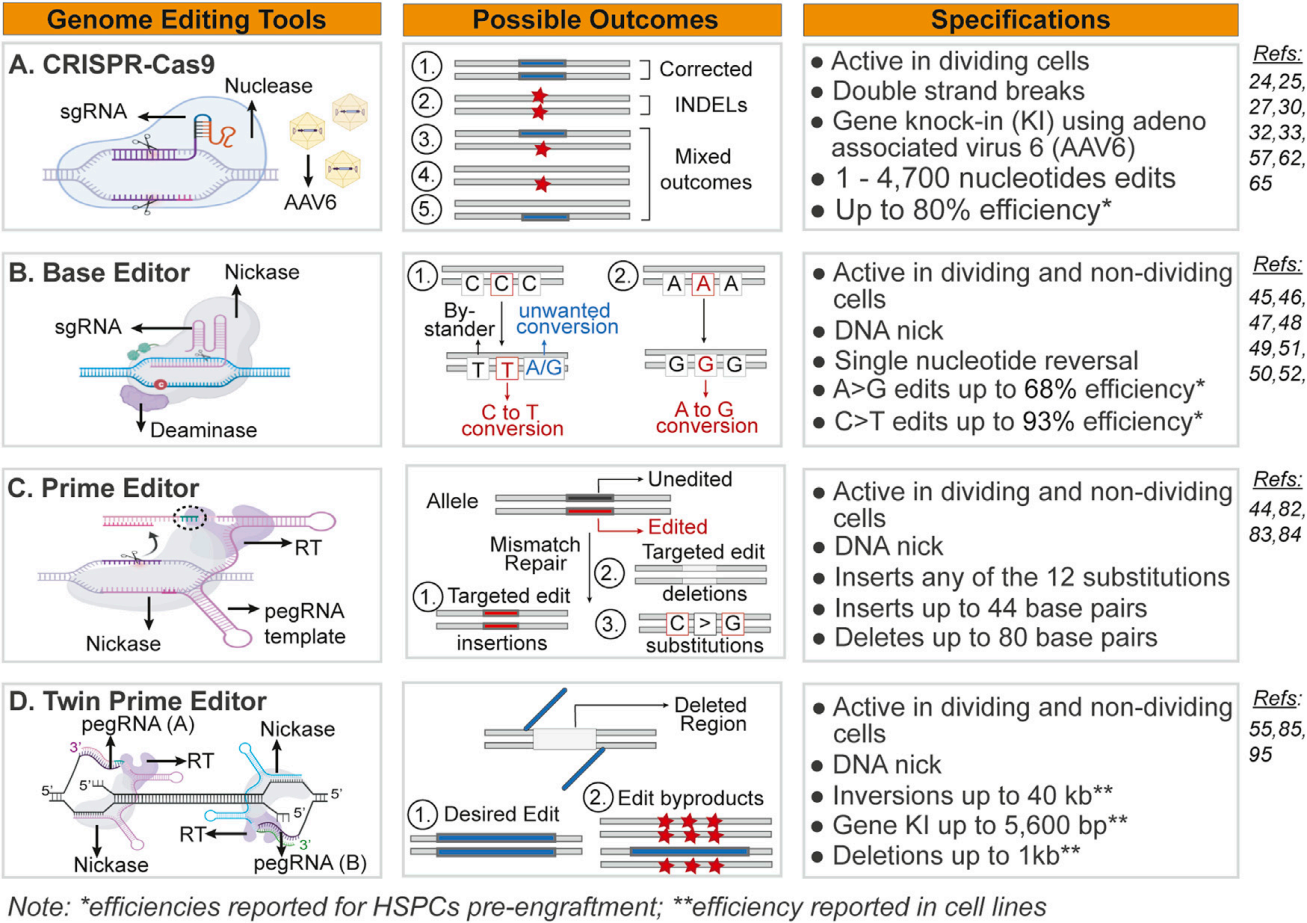
Overview of genome editing platforms discussed in this review. Schematics and description of the genome editing tools, genomic outcomes following editing and specifications for **(A)** CRISPR/Cas9-AAV6, **(B)** Base Editing (BE), **(C)** Prime Editing (PE), and **(D)** Twin Prime Editing platforms. sgRNA, single guide RNA; Nuclease, Cas9; Nickase, an engineered Cas9 nuclease with a single-stranded “nick” DNA cutting activity; AAV6, adeno-associated virus of serotype 6; INDELs, insertion and deletions; pegRNA, prime editing guide RNA; RT, reverse transcriptase; HSPCs, hematopoietic stem and progenitor cells.

Genome correction outcomes vary depending on the DNA template and repair pathway used to fix the Cas9-sgRNA-induced DSBs (Xue and Greene, 2021). In dividing cells, where homologous repair (HR) and the non-homologous end joining (NHEJ) DNA repair proteins are expressed, there are two primary editing outcomes (Figure 1A). When an exogenous DNA repair template carrying the desired DNA sequence modification is flanked by homology arms complementary to the DSB site, it acts as a template for HR. This corrective cassette is often delivered by an adeno-associated virus (AAV) with serotypes specific to the cell type of interest (e.g., AAV6 for HSPCs and T cells). The CRISPR/Cas9-AAV6 strategy was used to restore the endogenous expression of a mutated gene by inserting a therapeutic cassette that delivers a copy of a sequence (up to 4.7 kb) coding for a functional protein (Genovese et al., 2014; Hubbard et al., 2016; Schiroli et al., 2017; Pavel-Dinu et al., 2019; Goodwin et al., 2020; Cromer et al., 2021; Sweeney et al., 2021; Pavel-Dinu et al., 2022; Iancu et al., 2023).

In the absence of an exogenous HR-dependent corrective template, the repeated cycles of CRISPR/Cas9-sgRNA cutting coupled with NHEJ repair lead to nucleotide insertions and deletions (INDELs) at the site of repair (Rose et al., 2017) that likely disrupts the expression of the gene, generating a knockout (KO). Depending on the gene’s function, a KO allele could be therapeutic if it disrupts a dominant negative function (e.g., the *STING* gene in SAVI disease) (Fremond et al., 2021). However, it could also pose a therapeutic risk if the presence of INDELs induces a secondary disease (e.g., *HBB* gene and B-thalassemia) (Cromer et al., 2021). The limit of HR-based genome editing with AAV6 is up to 4,700 nucleotides, making it a popular correction approach for genes carrying hundreds of pathogenic variants spread throughout the coding sequence (Hirsch, 2015). However, this approach has several limitations. First, the process requires dividing cells, limiting the cell types amenable to such a genome editing approach (Xue and Greene, 2021). Second, delivery of the donor template to target cells depends on the availability of a viral vector with a serotype permissible to the modified cells. Third, a viral vector could trigger an immune response that could partially reduce the health of the cells being edited (Cromer et al., 2018; Schiroli et al., 2019). New technologies using viral vector-free delivery of the donor DNA template are being developed and hold promises (Shy et al., 2022).

Despite no major safety concerns have emerged in preclinical *in vitro* and *in vivo* studies, DNA editing with CRISPR/Cas9-AAV6 requires a careful evaluation of possible off-target sites, especially when those sites are present within genes affecting hematopoiesis. Currently, this evaluation is performed by a combination of methods, including *in silico* prediction and deep sequencing of *in vitro* edited healthy donors’ and patients’ derived HSPCs. Recent advancements in CRISPR/Cas9 technology have generated a new version of the Cas9 nuclease with significantly improved fidelity (Vakulskas et al., 2018), which has reduced and, in many cases, almost eliminated off-target site nuclease activity (Gomez-Ospina et al., 2019; Pavel-Dinu et al., 2019).

In addition to the unintended cutting activity, genotoxicity can be caused by chromosomal aberration typically induced by two or more guides or by using a single guide with additional homologous cut sites in the genome. However, in preclinical studies where the correction of a single defective gene relies on a single guide to introduce the desired genomic modification, the likelihood of introducing chromosomal aberrations is significantly reduced (Lattanzi et al., 2021; Cromer et al., 2022). To advance the preclinical studies for in-human-testing rigorous analyzes of the potential structural aberrations should therefore be performed using up-to-date technologies (Turchiano et al., 2021; Park et al., 2022).

The *ex vivo* editing of human HSPCs has been shown to trigger cellular response to CRISPR/Cas9-induced double-strand breaks and elicit an immune response to AAV6 containing a homologous template (Cromer et al., 2018; Allen et al., 2022; Ferrari et al., 2022). These responses could affect the stemness of the interventional products, especially when using patients’ HSPCs that have been exposed to prolonged inflammation *in vivo*. The preclinical studies using primary and secondary transplants in humanized mice show that edited HSPCs can sustain long-term engraftment and multilineage differentiation potential (Pavel-Dinu et al., 2019; Goodwin et al., 2020; Scharenberg et al., 2020), however, the frequency of edited HSPCs that persist long-term in humanized mice is diminished (Pavel-Dinu et al., 2019; Scharenberg et al., 2020). Depending on the disease indication (e.g., conditions where corrected HSPCs have a survival advantage over non-corrected ones) this decrease can still translate into a clinical outcome of disease correction. In other indications, the level of gene correction and frequency of edited HSPCs that need to persist long-term in the graft might need to be higher.

AAV6 delivery of the therapeutic cassette remains a promising and efficient approach. However, further improvements are necessary to reduce or eliminate potential toxicity from AAV6 exposure, an area of ongoing investigation (Scala et al., 2018; Ferrari et al., 2020; Sharma et al., 2021). In addition to AAV6, other viral delivery systems such as integrase-defective lentiviral vectors may provide certain benefits and be used for disease applications (Ferrari et al., 2022).

To address these limitations, increase the homogeneity of the genome editing outcomes and ensure the highest ratio of HR to INDELs, the genome editing toolbox has expanded to include new innovative tools—Base Editing (BE) (Komor et al., 2016; Jeong et al., 2020; Zeng et al., 2020), Prime Editing (PE) (Urnov, 2020; Nelson et al., 2022), and Twin Prime Editing (twinPE) (Anzalone et al., 2022; Choi et al., 2022) - that are active in non-dividing cells and are not reliant on double-strand breaks.

### Genome editing with a nick

Cas9 is a versatile nuclease: it can be engineered to introduce double-strand or single-strand nicks. It can also ferry protein subunits with gene regulatory functions (Qi et al., 2013; Gilbert et al., 2014; Anzalone et al., 2019; Pickar-Oliver and Gersbach, 2019). Three new genome editing platforms were recently engineered as protein chimeras by fusing the Cas9 nuclease to a deaminase, dubbed as Base Editing (BE), or linked to reverse transcriptase (RT), generating the Prime Editing (PE) and Twin Primer Editing (twin PE) strategies.

These newly developed genome editing platforms were designed to precisely edit DNA sequences independent of cell cycle status and without inducing DSBs in the mammalian genome. BE platform (Komor et al., 2016) can correct pathogenic variants and inactivate genes or cis-regulatory elements in HSPCs. Conversion of a C:G to a T:A base pair using cytosine BE (CBE) (Sakata et al., 2020) or the alternative reaction, T:A to C:G using adenine BE (ABE) (Gaudelli et al., 2017), is achieved using a catalytically dead or nickase Cas9-sgRNA complex fused to a deaminase (Figure 1B). Following DNA unwinding and complementary base pairing between the sgRNA and the targeted DNA sequence, the deaminase converts the targeted base located at a distance from protospacer (PAM): cytosine to uracil or adenosine to inosine, resulting in a C to T or an A to G change, respectively. Four generations of BE have been created to increase efficiency and improve safety (Komor et al., 2016; Komor et al., 2017). Further advancements have been implemented to broaden the targeting range using nickase Cas9 and deaminase variants (Gehrke et al., 2018), to recognize different protospacer adjacent motifs (PAM) (Hu et al., 2018; Nishimasu et al., 2018) or non-NGG PAMs (Chatterjee et al., 2018), and edit previously inaccessible genomic regions (e.g., methylated regions) (Wang et al., 2018), respectively.

Unlike BE platform, which allows for A>G, C>T, and C>G substitutions, PE was developed to ensure that all 12 possible base conversions—transversions and transitions—can be created. It is based on chimeric Cas9 nickase fused to reverse transcriptase (RT). It uses prime editing guide RNA (pegRNA) to serve as a sgRNA and a template for genomic modification without requiring a PAM sequence to mark the target site (Anzalone et al., 2019). The pegRNA moiety hybridizes with the nicked DNA strand, which acts as a primer for the RT reaction. The newly synthesized 3’ end of the DNA containing the desired edit invades the nearby DNA strand and primes the non-edited strand, thus introducing the desired DNA change in both strands (Figure 1C). The final products of PE-based genome editing range from single nucleotide changes to insertions (up to 44 base pairs) and deletions (up to 80 base pairs) at 5–50 base pairs away from the nick site in non-dividing cells (Anzalone et al., 2019). Although PE cannot modify thousands of nucleotides like a DBS-induced HR-based genome editing platform, it can correct an exon of a gene. We refer to the following reviews for a detailed discussion of the BE and PE mechanisms (Antoniou et al., 2021; Porteus et al., 2022).

TwinPE platform enables targeted integration of gene-size DNA fragments (Anzalone et al., 2022). Like, PE, twinPE uses a catalytically impaired Cas9 nickase. TwinPE has evolved to use two nickases, two RT enzymes, and two pegRNAs to specify the targeted genomic sequence and insert the desired edit (Figure 1D). Variations of this platform can generate sequence inversions and large knock-in (KI) (Anzalone et al., 2022; Choi et al., 2022; Zhuang et al., 2022). However, the latter is less effective than the CRISPR-Cas9-AAV6 platform.

In the long term, these innovations to the genome editing toolbox will expand the therapeutic options available to patients. Although, pre-clinical studies and further developments are still required to characterize the safety and efficacy of these platforms in primary cells, these new technologies hold great promises for advancing medicine.

## Applying DSB-based genome editing technology for correcting IEIs

### Genome editing for RAG2-deficiency, a primary immunodeficiency

The Recombinase Activating Genes 1 and 2 (*RAG1* and *RAG2*) are the natural genome-editing nucleases (Alt et al., 1992), whose activity is restricted to the lymphoid lineage and the G_0_/G_1_ cell cycle phase to prevent the risk of genotoxicity from promiscuous nuclease activity (Miyazaki and Miyazaki, 2021). Adaptive immunity depends on the function of these two nucleases to promote naturally occurring genome editing events necessary for normal development and the function of B and T cells. Through a process involving DNA cleavage and NHEJ-dependent DNA repair, the Variable (*V*), Diversity (*D*), and Joining (*J*) coding elements at the T cell receptor (TCR) and B cell receptor (BCR) loci rearrange to produce functional cell surface receptors. Productive and diverse rearrangements of the V-(D)-J give rise to functional antigen-specific receptors that ensure proper signals are received by T and B cells, respectively. These signals are required for normal development, elimination of lymphocytes reactive to self-antigens, and lymphocyte homeostasis. A diverse receptor repertoire also ensures that lymphocytes recognize a broad range of antigens and mount a normal immune response (Notarangelo et al., 2016). Over 200 unique pathogenic mutations (Corneo et al., 2001) are known to disrupt RAG1/2 proteins function resulting in *RAG* deficiency (Notarangelo et al., 2016; Tirosh et al., 2019), a PID with an incidence rate of 1:250,000 (Kwan et al., 2013; Dvorak et al., 2019). *RAG1/2*-deficient patients present with a broad spectrum of clinical manifestations that range from severe combined immunodeficiency (SCID) caused by biallelic null mutations (<5% recombinase activity) to atypical SCID (AS), delayed-onset combined immunodeficiency with granulomas and/or autoimmunity (CID-G/AI) and Omenn Syndrome (OS), and the latter being OS is caused by hypomorphic *RAG* variants that support residual (5%–30%) recombinase activity (Villa et al., 2001; Delmonte et al., 2018; Villa and Notarangelo, 2019; Delmonte et al., 2020).

Currently, the only definitive therapy for patients with *RAG1/2* deficiency is allogeneic hematopoietic stem cell transplantation (allo-HSCT), with survival rates as high as 90% for SCID patients when using HLA-matched donors (Castagnoli et al., 2019). For *RAG1/2*-OS, due to the severity exacerbated by the autoimmune manifestations, significant limitations are associated with allo-HSCT (Mazzolari et al., 2005). *RAG1/2* patients with AS or OS forms have a worse survival outcome than the typical SCID (Villa et al., 1999; Mazzolari et al., 2005).

Pre-clinical studies using *Rag1* (Pike-Overzet et al., 2011; Pike-Overzet et al., 2014; van Til et al., 2014) or *Rag2* (van Til et al., 2012) deficient mice models have shown variable therapeutic efficacy of the lentivirus (LV)-based gene addition approach in the HSPCs. For *Rag1*, the lymphoid lineage was only partially rescued by LV addition of a *RAG1* cDNA, with some mice showing signs of inflammation resembling OS (Pike-Overzet et al., 2011; van Til et al., 2014). For *Rag2*, the therapeutic efficacy of LV-based delivery of a codon-optimized (co*RAG2*) cDNA was more substantial but incomplete, with B cell immunity only partially restored (van Til et al., 2012). These findings suggest that autologous transplantation of LV-modified HSPCs overexpressing *RAG1/2* cDNAs might not recapitulate the endogenous levels of regulations and expression necessary to support healthy immune system development and function (van Til et al., 2012; van Til et al., 2014). In addition, ectopic expression of *RAG1/2* driven by exogenous promoters could lead to uncontrolled nuclease activity and translocation events. Results from an ongoing clinical trial for *RAG1* deficiency (NCT04797260) will offer more insight into the safety and efficacy of LV-based semi-random insertion of a nuclease into human stem cells’ genome.

Autologous transplantation of *RAG*1/2-deficient gene-corrected HSPCs could offer several benefits over the semi-random LV-gene addition strategy, including: preservation of endogenous regulation and expression, restriction of gene functions to the lymphoid lineage and the G_0_/G_1_ cell cycle phase, and avoiding the genotoxic risk from ectopic RAG1/2 activity.

We have recently developed and tested a genome editing approach to correct *RAG2* deficiency. In the first study (Gardner et al., 2021), we used induced pluripotent stem cells (iPSCs) and the CRISPR/Cas9-AAV6 platform to correct a *RAG2*-SCID mutation (c.831T>A) by inserting a functional codon-optimized (co) *RAG2* cDNA. Using an artificial thymic organoid (ATO) system that supports only *in vitro* T-cell differentiation, we showed that, unlike the *RAG2* ^null^ iPSCs, *RAG2*-corrected iPSCs demonstrated correction of the V(D)J recombination, thus overcoming the T-lymphocyte developmental blockage and differentiating into CD3^+^TCRαβ^+^ CD4/CD8 single positive lymphocytes, at levels comparable to healthy donor iPSCs. TCR sequencing of the derived T cells confirmed a polyclonal pattern of TCR alpha and beta chain rearrangement.

In a more recent publication (Iancu et al., 2023), another *in vitro RAG2-*SCID disease model was established. In this model, the *RAG2* gene was KO in human CD34^+^ cells using CRISPR/Cas9-AAV6 platform. The *RAG2* KO and control CD34^+^ cells were differentiated into T cells *in vitro*. The *RAG2*-deficient CD34^+^ cells failed to differentiate into CD3^+^ T cells, thus recapitulating the defect in *RAG2*-SCID patients. A very similar genome editing approach to ours for the correction of the *RAG2* gene was used. In this approach, a full-length codon-optimized *RAG2* cDNA was inserted at exon 1 of the endogenous *RAG2* gene, but unlike ours, it included a marker gene (*NGFR*) downstream of the therapeutic cassette, under the control of a constitutive promoter, allowing the selection of the edited cells. The resulting edited *NGFR*-purified CD34^+^ cells from healthy donors differentiate into CD3^+^ T cells with oligoclonal TCR repertoire diversity comparable to the controls. Importantly, similar results were obtained using *RAG2*-SCID patients’ CD34^+^ cells. Collectively, both *in vitro* studies support the feasibility of the CRISPR/Cas9-AAV6 editing platform to correct the *RAG2*-SCID.

We extended our studies *in vivo* (Pavel-Dinu et al., 2022), using healthy donors and patient-derived stem cells, to assess if the V(D)J activity can be reinstated in support of normal lymphoid lineage development (B cells in addition to T cells). Using a CRISPR/Cas9-AAV6 platform, we report 85% of alleles being genome-edited, of which 50% carried INDELs, and 30% were HR-modified. Since allo-HSCT studies show that a minimum of 20% donor chimerism is necessary to achieve clinical correction in *RAG*-SCID patients (Mazzolari et al., 2005), our reported levels of genomic modification surpassed the requirement for attaining clinically relevant levels of correction.

We further showed that the *RAG2*-corrected HSPCs derived from healthy donors and one *RAG2*-SCID patient could sustain long-term engraftment and multi-lineage differentiation following transplantation into immunodeficient NSG-SGM3 mice. Compared with uncorrected HSPCs, the patient-derived *RAG2*-corrected and engrafted HSPCs restored B cell development generating immunoglobulins M (IgM) expressing all 7-immunoglobulin heavy chain Variable gene families. We further detected T cells (CD3^+^, CD4^+^, CD8^+^) in the bone marrow and spleen of mice engrafted with *RAG2*-corrected HSPCs. The T cell receptors of patient-derived *RAG2*-corrected HSPCs showed comparable oligoclonal rearrangement patterns at the *TRA/TRD* locus compared to healthy donors and a similar pattern in TRAV and *TRAJ* gene usage.

Lastly, *in vivo* studies showed that the genome correction strategy shifted the predominant NK population from immature to mature natural killer (NK) cells phenotype. The immature NK cell phenotype was recently reported in patients with SCID caused by *RAG1/2* (Karo et al., 2014) and *NHEJ* deficiency (Dobbs et al., 2017). This is important to note because, in the absence of conditioning, this immature NK population with known heightened cytotoxic activity can attack the graft following allo-HSCT (Schuetz et al., 2014).

This is the first *in vivo* study that showed genome editing-based correction of V(D)J recombinase results in phenotypic correction of the lymphoid lineage defect in a typical *RAG2*-SCID patient-derived HSPCs, using the CRISPR/Cas9-AAV6 platform. This work provides the rationale for further development and testing of this strategy in the treatment and characterization of the autoimmune manifestations in *RAG2*-deficiency*.* The existing *in vivo* murine models of *Rag2* deficiencies (Mombaerts et al., 1992; Shinkai et al., 1992; Khiong et al., 2007; Marrella et al., 2007; Giblin et al., 2009; Ott de Bruin et al., 2016; Ott de Bruin et al., 2018) offer an optimal platform to assess the extent of the disease correction and safety, as a necessary step for advancing this approach to in human testing.

### Genome editing for *FOXP3*-deficiency, a primary immune regulatory disorder

Immune dysregulation polyendocrinopathy enteropathy X linked (IPEX) syndrome is a prototype monogenic autoimmune disease or PIRD caused by loss of regulatory T cell (T_reg_) function. IPEX syndrome is due to mutations in the *FOXP3* gene, a critical transcriptional factor that regulates and maintains the suppressive function of T_reg_ cells (Powell et al., 1982; Bennett et al., 2001; Wildin et al., 2001). Over 200 pathogenic variants span both protein-coding and non-coding regions, though usually not abrogating FOXP3 protein expression (Barzaghi et al., 2018; Gambineri et al., 2018). Loss of T_reg_ function due to *FOXP3* mutations leads to the loss of peripheral self-tolerance, expansion of autoreactive T cells, and the development of multiorgan autoimmunity (Bennett et al., 2001; Wildin et al., 2001; Fontenot et al., 2003; Borna et al., 2022a). IPEX typically manifests within the first year of life with type 1 diabetes, enteropathy, and eczema (Barzaghi et al., 2018; Gambineri et al., 2018). However, atypical forms of IPEX, including lupus-like manifestations or neuromuscular impairment, are becoming more frequently reported (Consonni et al., 2021; Rim et al., 2021). Current treatment options, including pharmacological immunosuppression and allogenic-HSCT, are only partially beneficial with limited outcomes (Barzaghi et al., 2018; Gambineri et al., 2018). Therefore, gene therapy is a great promise to address this unmet medical need.

**FIGURE 2:**
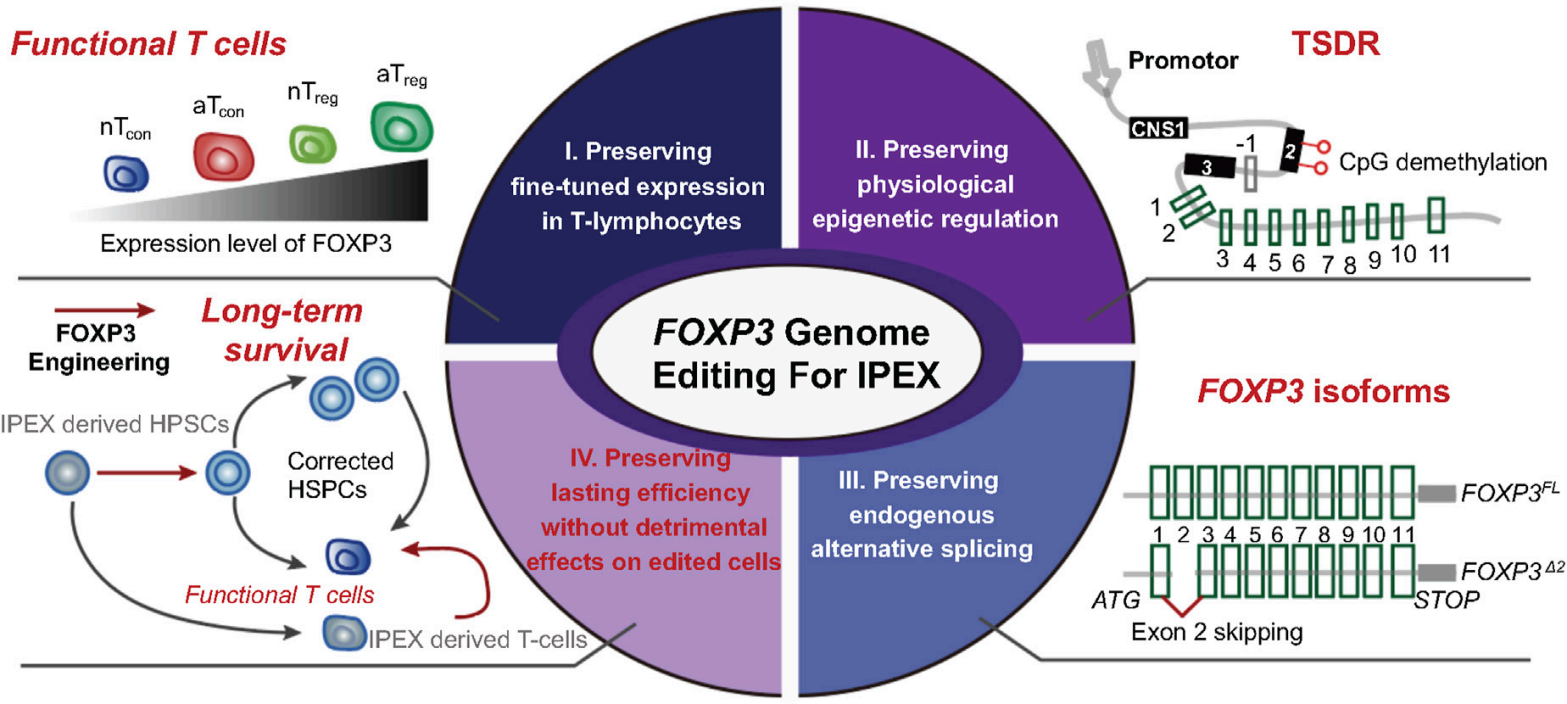
Challenges of FOXP3 genome editing in HSPCs for therapeutic purposes. Schematic representation of various features of the FOXP3 gene, which need to be preserved to achieve therapeutic safety and efficacy. nT, natural occurring regulatory or conventional T cells, aT, activated regulatory or conventional T cells, TSDR, Treg specific demethylated region, CNS, conserved non-coding sequence elements. Inspired from Figure 2, Porteus et al. (2022).

For an overview of the various gene therapy approaches to treat IPEX, please see our recent review (Borna et al., 2022b) and the open clinical trial (NCT05241444). Here, we focus on the CRISPR/Cas9 gene editing approach in HSPC. Like the *RAG2* gene, *FOXP3* gene expression and functions are tightly and differentially regulated in specific subtypes of CD4^+^ T cells. The most well-described regulatory components of the *FOXP3* gene are three conserved non-coding DNA sequence (CNS) elements. CNS1 plays a role in the generation of T_reg_ cells from effector T cells (T_eff_) in the periphery (Zheng et al., 2010). CNS2 contains a T_reg_-specific demethylation region (TSDR). The TSDR is demethylated only in T_reg_, to support high and stable FOXP3 expression (Floess et al., 2007; Polansky et al., 2008; Kressler et al., 2020). The TSDR is fully methylated in T_eff_, where FOXP3 expression is upregulated only transiently in response to TCR stimulation (Baron et al., 2007). Lastly, CNS3 is a pioneering element with a critical role in the induction of FOXP3 expression during T_reg_ development (Zheng et al., 2010). The optimal *FOXP3* editing strategy in HSPCs faces several challenges (Figure 2). It must preserve these regulatory mechanisms to achieve 1) physiologically high expression of the FOXP3 in T_reg_, 2) transient FOXP3 expression in T_eff_ upon TCR stimulation, and 3) prevent ectopic FOXP3 expression in other leukocyte subsets.

The consequence of constitutive and ectopic FOXP3 expression outside the T_reg_ cell compartment is well documented, using humanized mice. The immunodeficient mice were reconstituted with HSPCs transduced with a LV construct encoding a FOXP3 cDNA under the control of a constitutive promoter. In these mice, the human-derived T cells were hyporesponsive to TCR stimulation suggesting that ectopic FOXP3 expression may result in immunodeficiency (Santoni de Sio et al., 2017). Recently, we published a genome editing strategy to restore the expression of the *FOXP3* gene (Goodwin et al., 2020). Using CRISPR/Cas9-AAV6 platform, a cDNA coding for full-length *FOXP3* was inserted upstream of exon 1 of the endogenous *FOXP3* gene. The construct also contained an exogenous 3-phosphoglycerate kinase (PGK) promoter to drive the expression of a truncated form of Nerve Growth Factor Receptor ( Δ NGFR). Healthy donor T_reg_ cells edited with this construct expressed up to 50% of the levels of FOXP3 protein when compared to mock-treated T_reg_. The edited T_reg_ demonstrated suppressive capacity in co-culture suppression assay, although at the lower end range of the healthy donor T_reg_. In IPEX patient-derived T_reg_, the genome editing restored the suppressive function to near-normal levels.

The *FOXP3* edited HSPCs engrafted into immunodeficient mice demonstrated preserved multilineage differentiation potential. However, the number of T_reg_ in the spleen of these mice was significantly reduced, indicating that further optimization of the gene editing strategy is needed before going into the clinic. The reduced T_reg_ cell numbers *in vivo,* in the humanized mice, and the reduced suppressive capacity of the edited T_reg_ *in vitro* may be due to the lack of the additional *FOXP3* splice isoforms physiologically expressed in T_reg_. The alternative splicing of the endogenous *FOXP3* transcript gives rise to several splice isoforms. Of those, the full-length and the isoform lacking exon 2 ( Δ 2 FOXP3) (Mailer, 2018) are the most abundant. We found that *in vitro* transduction of both full-length and Δ 2 isoforms increase FOXP3 protein expression and suppressive function compared to double-transduced cells with the same isoform (Sato et al., 2021). This suggests that an optimal gene editing strategy for the *FOXP3* locus must preserve the gene’s splicing potential and its physiological expression and regulation (Figure 2). Overall, our work demonstrated the feasibility of the genome editing approach for re-establishing at least a partial T_reg_ cell function. This approach has broader applications to other PIRDs where the mutations in genes affect the function of T_reg_ and T_eff_ cells. Once the *FOXP3* gene correction strategy is optimized to address these limitations, the question remains whether the edited cells will have an *in vivo* selective advantage over the uncorrected ones. Additional preclinical work will be needed to determine if this genome editing platform is the optimal approach to restore *FOXP3* gene expression.

## Conclusion

Clinical application of genome editing is already a reality with different indications in medicine. Several approaches are in the clinic in oncology, mainly in *ex vivo* generated CAR-T. HR-mediated genome editing is a promising treatment for monogenic diseases of the blood and immune system. Ongoing trials for sickle cell and thalassemia begin to generate essential data for improving the current editing protocols. GraphiteBio gave a recent press release on the slow engraftment of the gene-corrected product in the first patient treated as part of the CEDAR clinical trial NCT04819841. The field looks forward to more details behind this report so that a better understanding and potential solutions might be developed.

A combination of newborn screening and genome editing in autologous HSPCs could provide a one-time definitive therapy for several IEIs. Moreover, autoimmunity and inflammation could also benefit from the knock-in genome editing strategy to modulate regulatory genes (e.g., *FOXP3*) in specific cell subsets (e.g., conventional T cells) (Honaker et al., 2020). In addition, new approaches are being developed to eliminate and change the TCR specificity of a T cell product to modulate immune responses (CAR-T_reg_ genome editing).

The editing platforms will continue to advance, allowing simultaneous editing of multiple genes, as in the case of patients diagnosed with multiple Variants of Unknown Significance (VUS).

Optimizing DSB-independent and viral-free genome editing platforms could be a significant step forward for IEIs. Overall, the CRISPR/Cas9-based editing technology provides hope for a new generation of precision medicine treatments over a wide range of indications, provided it is ethically performed to overcome incurable diseases. The big challenge for the future will be to make these therapies accessible to all patients in need (Nature Medicine, 2021).
